# Image Analyzer Study of the Skin in Patients With Morbid Obesity and Massive Weight Loss

**Published:** 2015-01-23

**Authors:** Khaled Sami, Ahmed Elshahat, Manal Moussa, Alaa Abbas, Amr Mahmoud

**Affiliations:** ^a^Plastic Surgery Department, Faculty of Medicine, Ain Shams University, Cairo, Egypt; ^b^Histology Department, Faculty of Medicine, Ain Shams University, Cairo, Egypt; ^c^Surgery Department, Faculty of Medicine, Ain Shams University, Cairo, Egypt

**Keywords:** image analyzer, collagen, massive weight loss, obesity, skin laxity

## Abstract

**Objective:** Studies in literature on skin of patients with massive weight loss are limited and somehow conflicting in their results. The aim of this study was to quantitatively assess the skin change after massive weight loss. **Method:** This study was performed on 30 patients from whom skin biopsies were taken from the skin excised during operations, divided into 3 main groups. The first included patients who were undergoing cosmetic contouring procedures without history of massive weight loss. The second included patients with morbid obesity, who were undergoing bariatric surgery. The third included patients with massive weight loss submitted to cosmetic contouring procedures after stable weight reduction for 6 months. Biopsies were taken from the skin for histological testing. Hematoxylin and Eosin, Mallory, and Aldehyde fuchsin Stains were used to assess the skin collagen and elastic fibers. For quantitative assessment, the Image Analyzer System (Leica Q 500 MC program) was employed. Tensile tests were applied to skin samples using (Instron 5500R) Universal testing machine to measure the skin tensile strength to determine the maximum stress (Burst strength) that skin can induce before damage. **Results:** Collagen was significantly thinner in massive weight loss group in both papillary and reticular dermis and significantly less dense in reticular dermis with damage to the elastic fiber network. **Conclusion:** The skin of the patients with massive weight loss is weak due to lower density and thickness of collagen fibers and damage to its elastic fibers.

In developed Western culture, a lean body is considered to reflect good self-control and is regarded as attractive, healthy, and socially acceptable. Obesity is a stigmatizing disorder, especially among women, which may explain why women predominate in seeking treatment as they usually disrespect their body shape.^1^

Obesity triggers quite a few dermatologic aberrations, affecting skin itself (barrier function), adnexae (sweat and sebaceous glands, hair follicles), vascularity, lymphatics, microcirculation, and response to trauma (wound healing). Recurrent infectious troubles, hyperpigmentation, edema and ulcerations, striae, cellulitis, and other disorders are not uncommon either.^2^

The weight loss following bariatric surgery or diet is usually accompanied by an improvement in body image, and in the majority of operated subjects these feelings remain at a stable level in the long-term.^1^

In clinical practice, however, in spite of highly satisfactory weight loss and maintenance, some individuals still disrespect their body image and seek body-contouring procedures to improve their physical appearance. Skin and soft tissue redundancy following massive weight loss is considered unsightly. Some contraction of the skin envelope can be expected for about 1 year after a stable weight is reached, but little change is likely to occur thereafter and plastic surgical procedures may be necessary to satisfy patients’ needs and restore body image.^3^

The etiology of skin laxity after rapid weight loss is inadequately understood. It usually occurs because of damage of collagen and elastin, which allows for no skin retraction after weight loss.^4^

Macromolecular components of connective tissue are dependent on age, gender, nutritional imbalances, hormones including those of pregnancy, use of certain drugs and chemicals, and assorted genetic and environmental factors, including prolonged exposure to sunlight and other forms of radiating energy.^2^

There is a common universal belief among plastic surgeons that skin quality is impaired in patients with massive weight loss. However, this hypothesis has yet to be tested.^5^

While previous studies have reported patients with massive weight loss who undergo contouring procedures are at an increased risk for healing complications such as hematomas and seromas, another undesirable outcome that cannot be easily measured is skin relaxation after contouring procedures for patients with massive weight loss. The untested belief is that obesity and massive weight loss result in skin laxity after surgery due to damaged skin, those patients with massive weight loss have skin with miserable elasticity, thinned strength layers like the dermis and epidermis, and poor collagen quality.^6^

Obesity and massive weight loss represent a scientific new field for investigation. While studies have investigated, clinical outcomes in bariatric and postbariatric body-contouring populations, it is time to look on a more microscopic level to understand the effects of both obesity and massive weight loss on skin and wound healing, and to assess the possible causes of skin laxity following contouring procedures.

## PATIENTS AND METHODS

This study was performed on 30 patients from whom skin biopsies were taken from the skin excised during operations, divided into 3 main groups.

The first included patients who underwent cosmetic contouring procedures without history of massive weight loss.

The second included patients with morbid obesity, who underwent bariatric surgery.

The third included patients with excess weight loss—more than 30% of total body weight or 50% of excess weight^5^ submitted to cosmetic contouring procedures after stable weight reduction for 6 months.

Inclusion criteria: Age 18 to 65 years submitted to elective operations.

Exclusion criteria: Sepsis, shock, coma, previous plastic surgery intervention, Cushing disease, use of corticosteroids for allergy, inflammatory bowel disease or other condition, clinically diagnosed malabsorption or protein calorie malnutrition, pregnancy or lactation, any hospitalization in the last 2 months, and refusal to participate in this study.

Biopsies were taken from the skin for histological testing. After collection, subcutaneous adipose tissue was excised from the skin samples. Then skin samples were placed in 10% formalin/phosphate buffer. Biopsies were then embedded in paraffin before slicing. Each biopsy was sectioned at 3- to 5-μm intervals, creating a number of thin slices, which were transferred onto glass slides. The samples were then stained using the following:

1- Hematoxylin and eosin stain: It was used to examine the 3 layers forming the skin in all skin sections.
2- Mallory's trichrome stain: This staining technique was used to reveal collagen.
3- Aldehyde fuchsin stain: It was used to stain elastic fibers.

For quantitative assessment of tissue fiber density, the Image Analyzer System (Leica Q 500 MC program) was employed. Briefly, microscopic images (×400) in 10 consecutive fields were digitized to assess area percentage of collagen fibers and collagen fiber thickness in both papillary and reticular dermis.

Tensile tests were applied to skin samples using (Instron 5500R) Universal testing machine with 1 kN piezoresistive load cell to measure the tensile load and an acquisition frequency of 5000 Hz. This type of instron has a self-calibration, zero adjusting, and automatic balance, which are done before testing; this testing instrument is accompanied by a highly reliable system for evaluating the mechanical properties.

This study was used to measure the skin tensile strength to determine the maximum stress (Burst strength) that the skin can induce before damage with burst strength recorded as the peak value in newtons (Ultimate tensile strength [UTS] in MPa, often shortened to tensile strength or ultimate strength, is the maximum stress that a material can withstand while being stretched or pulled before failing or breaking).

## RESULTS

The study was successfully performed on 30 patients; in the first group (the patients with massive weight loss) the mean patient age in years was 39.2 ± 9.86 and all had no associated comorbidities as mentioned in exclusion criteria, while in the normal weight group the mean age of the patients was 39.3 ± 7.73, and in the last group (the morbid obese) the mean age was 37.8 ± 7.05. Samples were taken in a direction parallel to Langer's lines except in the morbid obese were perpendicular to the lines so as not to add harm to the patient with a new wound as the incision is usually vertical in direction. All skin samples were taken during operations with no harm applied to the patients during the procedure. No major complications were seen in patients during or after the procedures, and all were fully conscious postoperatively and in good health. Histological studies were successfully done within time of sample taking and results were statistically analyzed as will be seen.

### Collagen fiber assessment in massive weight loss group

**Figure 1:**
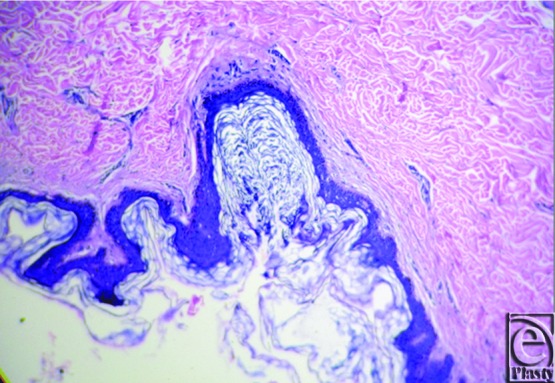
Hematoxylin and eosin stain showing thin irregular epidermis with very thick keratin layer and basket weave appearance.

**Figure 2:**
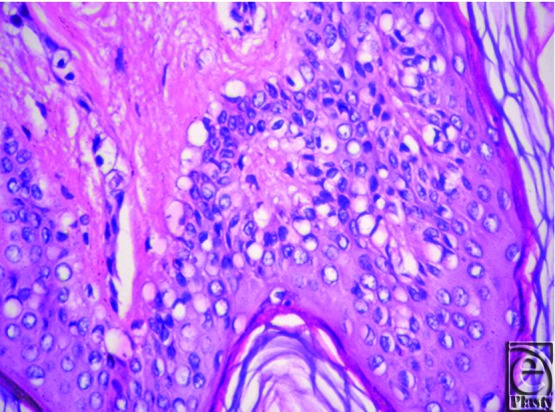
Hematoxylin and eosin stain showing atypical keratinocytes with highly vacuolated cytoplasm and deeply stained thin nuclei.

The epidermis in this group was markedly thin, with marked thickness irregularities, marked folding and corrugations, and thick keratin layer that reaches the same skin thickness in some samples with epidermal multiple irregular sacules, indicating keratin trapped within the epidermis; many keratinocytes are atypical with markedly vacuolated cytoplasm, and shrunken deeply stained thin stretched nuclei and rupture of cells in the granular cell layer could also be seen (Figs 1 and 2).

The main skin changes in these patients could be seen in the dermis regarding both collagen and elastic fibers.

**Figure 3:**
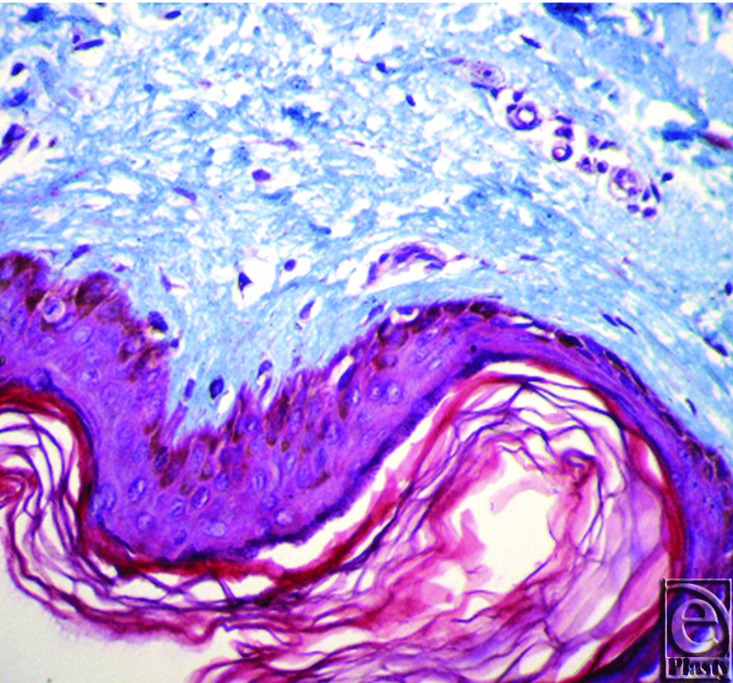
Mallory stain showing moth eaten appearance of collagen fibers with loss of fibrillary content.

**Figure 4:**
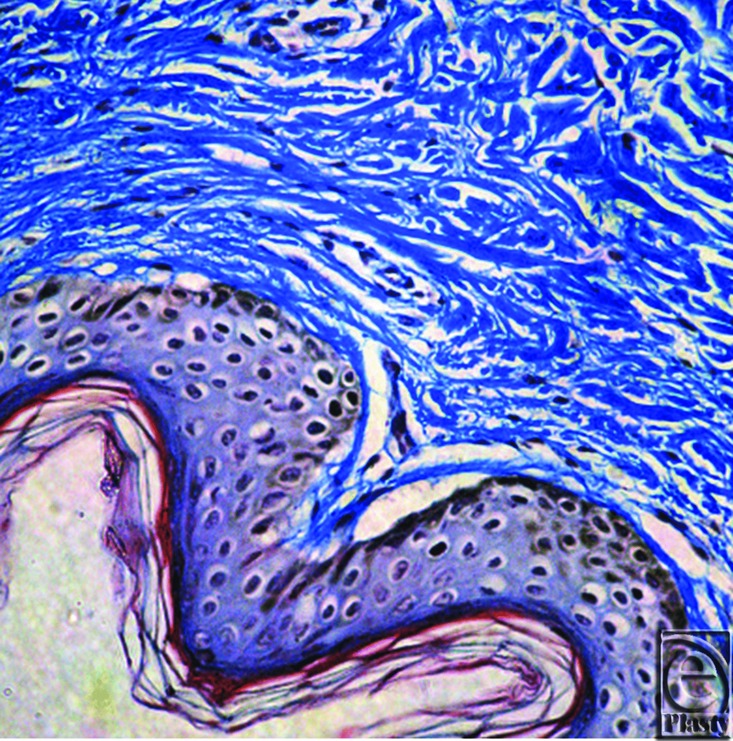
Mallory stain showing dense collagen bundles running parallel to the dermoepidermal junction (fibrosis).

The normal thin branching collagen bundles in the papillary dermis (Figs 3 and 4) is not seen with loss of the fibrillary content of collagen with many areas showing moth eaten appearance and areas of homogenous color where no collagen fibers could be seen.

In some areas, collagen form dense fibers that run horizontally parallel to the dermoepidermal junction indicating fibrosis. From these findings, it is noted that severe damage to the papillary dermis collagen is seen.

**Figure 5:**
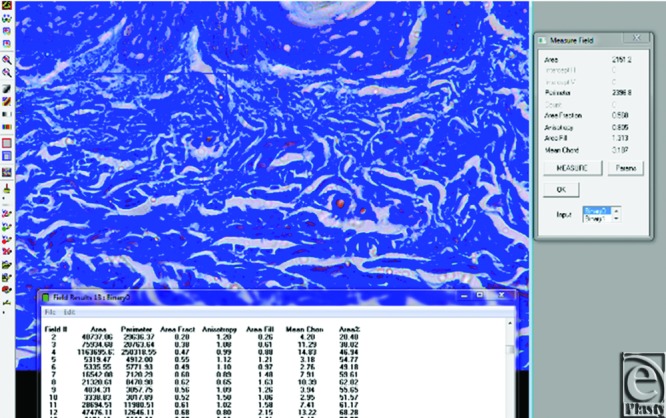
Image analyzer measuring collagen fiber density and thickness.

By image analyzer (Fig 5), collagen density in the papillary dermis was 56.22% ± 6.74% with collagen fiber thickness (diameter) 1.19 ± 0.38 μm.

Reticular dermis collagen is seen to lose its normal shape, arrangement, and concentration. The normal shape of the long thick branching wavy interdigitating fibers running parallel to the surface of the skin is totally lost with appearance of short thin cut fibers with areas of moth-eaten appearance of collagen bundles and homogenous areas where no definite fibers could be seen at all.

**Figure 6:**
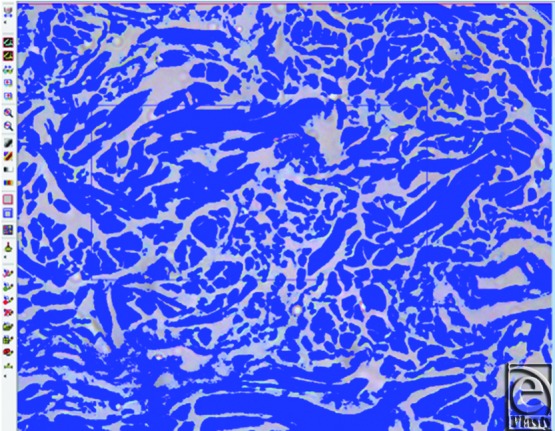
Image analyzer of reticular dermis showing loss of the fibrillary content of collagen.

Collagen density in the reticular dermis was 59.94% ± 2.42% with collagen fiber thickness (diameter) 5.16 ± 1.66 μm (Fig 6).

### Collagen fiber assessment in normal weight group

In this group of patients, histological changes affecting different layers of the skin variably could be seen, but these changes are not severe as seen with the massive weight loss group, and the skin still shows the normal anatomy with areas of normal collagen and elastic fibers compared to the other group.

The epidermis shows normal thickness and appearance in most of the samples, while few showed mild to moderate thickness irregularities with some atypical keratinocytes with vacuolated cytoplasm and dispersed chromatin and thick keratin layer, the same findings seen in the massive weight loss samples but in a mild degree.

Papillary and reticular dermis in this group show many changes in collagen fibers, but these changes are not severe and appear in some areas and skip other ones, and the normal shape of collagen fibers, such as the fine branching fibers in the papillary dermis or the long thick branching wavy interdigitating fibers in the reticular dermis, is still seen in most samples.

In some areas, there is loss of the fibrillary content of collagen, disorganization of fibers, and dense fibers parallel to the dermoepidermal junction denoting fibrosis. But although these changes are close to those found in the massive weight loss group, they are mild to moderate and collagen fibers can still be seen in normal shape, thickness, and density in many areas of the skin.

Collagen density in the papillary dermis was 53.47% ± 8.53% with collagen fiber thickness (diameter) 1.78 ± 0.24 μm, while collagen density in the reticular dermis was 67.12% ±6.21% with collagen fiber thickness (diameter) 9.32 ± 2.04 μm.

### Collagen fiber assessment in morbid obese group

In this group, the skin was seen to be more or less normal in most aspects compared to normal skin histology in literature. Epidermis was slightly stretched but with good regular thickness and normal healthy keratinocytes. Increased number of melanocytes was noticed in multiple samples. Atypical cells that were seen in the other groups are not seen.

The papillary dermis showed normal thin branching collagen fibers with normal dense well-formed dermoepidermal junction, while reticular dermis showed long thick branching wavy interdigitating fibers in almost all samples. The loss of fibrillary content and fibrosis seen in other groups is not seen here. Only few samples showed edema in the epidermis and dermis.

What is seen to be characteristic is the presence of many very large fat cells inside the reticular dermis, with very large fat cells seen in the connective tissue around sweat glands, blood vessels, and nerves.

Collagen density in the papillary dermis was 56.41% ± 5.55% with collagen fiber thickness (diameter) 2.19 ± 0.43 μm, while collagen density in the reticular dermis was 68.72% ± 3.67 with collagen fiber thickness (diameter) 9.03 ± 1.52 μm.

### Elastic fiber assessment in massive weight loss group

Elastic fiber arrangement is lost completely in the papillary dermis (normally thin elaunin fibers in the papillary dermis are seen running in a complete layer parallel to the dermoepidermal junction with fine oxytalan fibers extending perpendicular to the skin), while long thin branching elastic fibers are seen in the reticular dermis.

**Figure 7:**
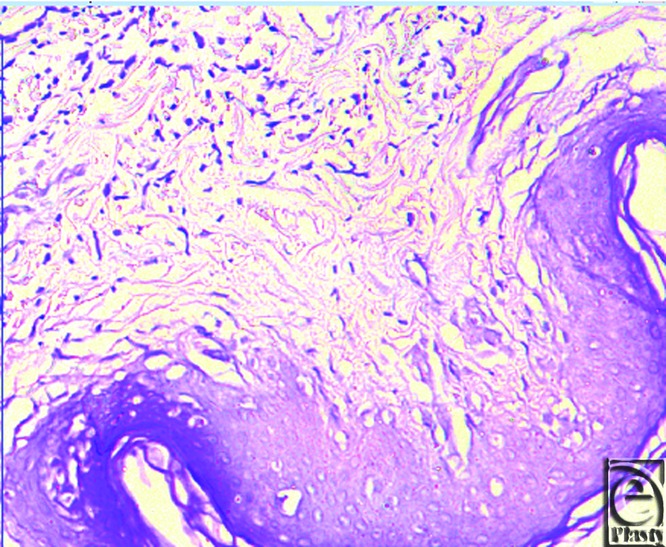
Aldehyde fuchsin stain showing loss of elaunin and oxytalan fibers in the papillary dermis with short fragmented elastic fibers in the reticular dermis.

In this group of patients, there is complete loss of the elaunin and oxytalan fibers from the dermis; they may be seen in small areas but even though are scanty and disorganized. The reticular dermis shows short fragmented elastic fibers, and in some areas no fibers could be seen at all with increased elastin protein deposition (elastosis) (Fig 7).

### Elastic fiber assessment in normal weight group

Elastic fibers in this group were almost normal, with complete layer of elaunin fibers run parallel to the dermoepidermal junction, from which they extend fine network of oxytalan fibers perpendicular to the skin. Long thin branching elastic fibers are seen in the reticular dermis with no evidence of elastosis.

Although some samples showed mild changes like incomplete layer of elaunin or oxytalan fibers and some elastin protein deposition, they were mild and insignificant and these are considered a normal variant.

### Elastic fiber assessment in morbid obese group

Elastic fibers are seen to be affected in most samples. Although the elaunin, oxytalan, and elastic fibers are seen in all cases, many samples show areas of disorganized fibers, the elaunin layer is seen incomplete in some areas and disappear with the oxytalan fibers in others, and some elastic fibers appear short and fragmented. Overall, we can detect abnormalities but not that much as in the massive weight loss group.

Statistical analysis of data obtained from our histological study (Table 1) showed that there was a high statistical significance between study groups except in one item, which is the collagen density in the papillary dermis where data showed no statistically significant differences between the three groups.

Collagen fiber thickness in the papillary dermis was the only statistically significant difference between the normal weight and morbid obese groups as it was lower in the normal weight group, and of course there was a statistically significant difference between massive weight loss group and the other 2 groups as it is seen to be much lower in the massive weight loss.

Collagen fiber density in the reticular dermis of massive weight loss group was significantly lower than the other 2 groups, with no statistically significant differences between the morbid obese and normal weight groups.

Finally collagen fiber thickness in the reticular dermis was markedly lower in the massive weight loss group (much affected than other variants) with no statistically significant differences between the other 2 groups.

The results of the biomechanical tests showed some degree of variation in the results of samples of the same patients and in-between patients. Difficulties encountered are mostly due to the anisotropic nature of the skin. The elastin and collagen fibers along the Langer's lines are more stretched than those perpendicular to the lines. Therefore, the extensibility of skin is lower (and thus a higher stiffness) in the direction of these lines. These variations are usually expected in testing soft biological tissues like human skin.

For each tensile test, the UTS was calculated by dividing the force applied by the undeformed cross-sectional area of the specimen.

Results of the tests are summarized in Table 2. The UTS of the massive weight loss group was remarkably lower than that of the other groups (13.31 ± 2.17 MPa) compared to (23.93 ± 3.38 MPa) in normal weight group and (24.98 ± 2.50 MPa) in morbid obese group.

## DISCUSSION

A common belief among plastic surgeons is that skin quality is much affected and impaired in patients with massive weight loss; this could be seen in different aspects starting from the simple clinical examination of the skin that shows thin inelastic wrinkled highly folded dry skin.^5^ During contouring surgeries, it is also noted how skin is much thinner in thickness and dermoepidermal integrity is clinically seen much affected and in many cases the epidermis could be peeled easily from the underlying dermis.^3^

Following contouring procedure, it is also noted that postoperative complications are commonly higher in these patients ranging from delayed healing, wound disruption and ending up with skin redundancy following surgery especially when compared to normal weight persons submitted to the same surgeries.^7^

Despite all these clinical observations, studies in literature on skin of patients with massive weight loss are scanty, limited, and somehow conflicting^6^ and up till now skin changes that occur with massive weight loss are not fully addressed and understood.

Because many factors may affect the skin in patients with massive weight loss such as the degree of morbid obesity, the rate of weight loss, the procedures performed to achieve weight loss, the nutritional status of these patients before and after weight loss, and changes to the skin that may have occurred other than weight changes like repeated pregnancies in females, the study of skin changes in patients with massive weight loss is a little bit difficult and needs more to address the effect of each variant on the skin; of course, that should be done after addressing skin changes occurring with massive weight loss as a first step to understand.

Orpheu et al^5^ compared skin in the epigastrium and hypogastrium of patients with massive weight loss and with never obese persons undergoing cosmetic contouring procedures and found that skin of patients with massive weight loss was depleted in collagen both in the epigastrium and hypogastrium, while elastic fibers where increased in both. In their study, no detailed information were mentioned regarding changes in different skin layers, no information could be taken about collagen fiber shape, orientation, thickness, and percentage of collagen in the skin dermis. Light et al^8^ found almost the same results as did Orpheu et al.^5^

When comparing the results of the biomechanical studies on normal human skin in literature, it can be seen that there is much variation between authors. This is usually expected when applying these studies to biological tissues like human skin. First because of the biological differences between subjects, second because of the anisotropic nature of the skin itself, and third because of the different methods and devices used in testing.

Very little information in literature was found regarding biomechanical studies on skin of patients with massive weight loss. Choo et al^6^ performed a biomechanical and histological study to compare samples from medial and lateral abdominal skin between patients with massive weight loss and those with normal weight undergoing cosmetic contouring procedures, and they stated that there is no difference in tensile strength between abdominal skin of patients with massive weight loss and those with normal weight in their study, and they even mentioned that they found that the medial abdominal skin of patients with massive weight loss was stronger than control skin from the same area in both vertical and horizontal directions. They also stated that skin of patients with massive weight loss was thicker than that of normal weight group with increased thickness of both epidermis and dermis with thicker collagen fibers.

In this study, a histological study was applied to patients with normal weight, morbid obesity, and massive weight loss aiming not to miss changes that may occur with morbid obesity as well as massive weight loss. We found that morbid obesity did not affect the skin quality as it appeared almost normal with good collagen fiber thickness and density. The results were clear in showing that skin of patients with massive weight loss is much weaker and less resistant; it lacks the integrity of epidermis and dermis; the epidermis was significantly affected with marked thinning, irregularities, and atypical cells with obvious changes to the dermoepidermal junction; the collagen fibers are seen damaged in different degrees with lack of normal shape, distribution, and even density mainly in the reticular dermis with marked decrease in collagen fiber diameter; and areas of fibrosis could be seen obviously in the dermis. Elastic fibers were damaged and even absent in many subjects.

Statistical analysis of data obtained from the image analyzer showed that there was a high statistical significance between study groups especially with the massive weight loss group compared to the other groups.

In the biomechanical study, we found that the skin of patients with massive weight loss is much weaker and less resistant to breakage than the other groups with statistically significant difference in UTS (13.31 ± 2.17 MPa) compared to (23.93 ± 3.38 MPa) normal weight group and (24.98 ± 2.50 MPa) morbid obese group.

What in addition was noted is that no findings were identical in any samples obtained; each patient shows unique characteristics of his own skin that cannot be seen in any other person as if it is a tissue print.

Finally, we can say that the results support the clinical observations that the skin quality of patients with massive weight loss is impaired. In addition, this study gives a picture of the changes that occur in the process of morbid obesity and massive weight loss.

## CONCLUSION

Morbid obesity did not affect the skin quality as it appeared almost normal with good collagen fiber thickness and density, and only the elastic fiber network was impaired most probably due to excessive stretching applied to the skin. The results were clear in showing that skin of patients with massive weight loss is much weaker and less resistant, and it lacks the integrity of epidermis and dermis with highly destructed collagen and elastic fiber networks.

This study is a step to address skin changes associated with morbid obesity and massive weight loss, and further studies are needed in this field of research on skin of patients with massive weight loss to correlate skin changes with the possible causes and outcomes and to help reach the best options in treatment of these patients clinically and surgically to obtain the best outcomes and decrease the rate of complications.

## Figures and Tables

**Table 1:** Statistical analysis of the histological study tests

	Group								
	Massive weight loss (1)		Normal weight (2)		Morbid obesity (3)		Analysis of variance		
	Mean	SD	Mean	SD	Mean	SD	*P*	Significance	Post hoc test
Collagen density in papillary dermis	56.22	6.74	53.47	8.53	56.41	5.55	.59	NS	
Collagen density in reticular dermis	59.94	2.42	67.12	6.21	68.72	3.67	<.001	HS	1 vs 2* 1 vs 3*
Thickness (diameter) of collagen fibers in papillary dermis	1.19	0.38	1.78	0.24	2.19	0.43	<.001	HS	1 vs 2* 1 vs 3* 2 vs 3*
Thickness (diameter) of collagen fibers in reticular dermis	5.16	1.66	9.32	2.04	9.03	1.52	<.001	HS	1 vs 2* 1 vs 3*

*Highly significant post hoc test.

**Table 2:** Statistical analysis of biomechanical tests

	Group								
	Massive weight loss (1)		Normal weight (2)		Morbid obesity (3)		Analysis of variance		
	Mean	SD	Mean	SD	Mean	SD	*P*	Significance	Post hoc test
Ultimate tensile strength in MPa	13.31	2.17	23.93	3.38	24.98	2.50	<.001	HS	1 vs 2* 1 vs 3*

*Highly significant post hoc test.

## References

[1] Chandawarkar R-Y (2006). Body contouring following massive weight loss resulting from bariatric surgery. Adv Psychosom Med.

[2] Yosipovitch G, DeVore A, Dawn A (2007). Obesity and the skin: skin physiology and skin manifestations of obesity. J Am Acad Dermatol.

[3] Eisenberg D, Duffy A-J, Bell R-L (2006). Update on obesity surgery. World J Gastroenterol.

[4] Aly A-S, Cram A-E, Chao M (2003). Belt lipectomy for circumferential truncal excess: the University of Iowa experience. Plast Reconstr Surg.

[5] Orpheu S-C, Coltro P-S, Scopel G-P (2010). Collagen and elastic content of abdominal skin after surgical weight loss. Obes Surg.

[6] Choo S, Marti G, Nastai M, Mallalieu J, Shermak M-A (2010). Biomechanical properties of skin in massive weight loss patients. Obes Surg.

[7] D’Ettorre M, Gniuli D, Iaconelli A, Massi G, Mingrone G, Bracaglia R (2010). Wound healing process in post-bariatric patients: an experimental evaluation. Obes Surg.

[8] Light D, Arvanitis G-M, Abramson D, Glasberg S-B (2010). Effect of weight loss after bariatric surgery on skin and the extracellular matrix. Plast Reconstr Surg.

